# Clinical Value of Implantable Loop Recorders in Syncope of Unclear Aetiology: A Single-Centre Experience

**DOI:** 10.7759/cureus.92580

**Published:** 2025-09-17

**Authors:** Mamoon Ahmed, Joseph Morris, Sree M Bommireddi

**Affiliations:** 1 Internal Medicine, Northern General Hospital, Sheffield, GBR; 2 Cardiology, Manchester Royal Infirmary, Manchester, GBR

**Keywords:** arrhythmia, cardiac monitoring, diagnostic yield, implantable loop recorder, syncope

## Abstract

Background

The aetiology of syncope can be challenging to undermine because of its sporadic nature. Implantable loop recorders (ILRs) are recommended to be used early in undetermined syncope to establish a symptom-rhythm correlation if cardiac syncope is suspected. The real-world diagnostic yield of ILRs is not well established.

Purpose

This study aimed to evaluate the diagnostic yield of ILR devices in patients with undetermined syncope.

Methods

This single-centre, retrospective study reviewed the electronic medical records of all patients who underwent ILR insertion for syncope from January 2017 to June 2022. Abnormal rhythm strips on ILRs were reviewed by a cardiac physiologist and cardiology consultant to determine their significance. The primary outcome was the total number of cardiac implantable electronic devices (CIEDs) inserted due to ILR findings. Secondary outcomes included (1) a comparison of baseline characteristics between the group of patients who had an arrhythmia detected on ILRs and patients who had no detected arrhythmia and (2) the total number of clinically significant events (CSEs), defined as symptomatic arrhythmia, asymptomatic arrhythmia deemed clinically related to the patients' history of syncope, and the absence of arrhythmia when the patient experienced syncope. Complications of ILR insertion were recorded. Given that normality was not assumed, a non-parametric analysis was utilized to compare baseline characteristics.

Results

A total of 215 patients were included in this study, of which 43 (20%) had a CIED inserted due to ILR findings. Baseline characteristics were similar between the group of patients who had an arrhythmia detected on ILRs and patients who had no detected arrhythmia, except for age. One hundred three (47.9%) of the patients had CSEs. Symptomatic arrhythmias were the most common CSEs, out of which a sinus pause of greater than six seconds was the most prevalent arrhythmia. There were no complications that required early explant of the ILR in this cohort.

Conclusion

ILRs detected a clinically significant arrhythmia and the absence of an arrhythmia in 92 (42.7%) and 11 (5.1%) patients with undetermined syncope, respectively. ILR is a valuable diagnostic tool in this cohort of patients. Further studies looking at its cost-effectiveness would be beneficial.

## Introduction

The aetiology of syncope is often challenging to determine due to its transient and variable presentation. It can be due to a broad spectrum of potential causes ranging from benign neurocardiogenic mechanisms to life-threatening arrhythmias. Conventional diagnostic approaches, including electrocardiogram (ECGs), Holter monitoring, echocardiography, and tilt table testing, often fail to capture the event or establish a definitive cause because of the sporadic nature of certain syncopal presentations [1].

Implantable loop recorders (ILRs) address this limitation by enabling prolonged rhythm monitoring, correlating patient symptoms with cardiac activity at the time of syncope. International guidelines recommend the early deployment of ILRs in patients with recurrent unexplained syncope, particularly when a cardiac aetiology is suspected and standard evaluations are inconclusive [2,3], given the potential for timely diagnosis to significantly influence therapeutic decisions. However, despite these recommendations, the real-world diagnostic yield and effectiveness of ILRs remain variably reported, and their utility in routine clinical practice continues to be an area of ongoing investigation.

This article was previously presented as a meeting abstract at the 2023 European Society of Cardiology Conference on August 27, 2023 [4].

## Materials and methods

This was a single-centre, retrospective observational study conducted at Salford Royal Hospital, Northern Care Alliance NHS Foundation Trust, Salford, UK. We reviewed the electronic medical records of all consecutive patients who underwent ILR insertion for the evaluation of syncope between January 2017 and June 2022. Patients were included if the indication for ILR implantation was unexplained syncope after initial diagnostic assessment and workup. Patients who were under 18, were unable to give consent, have faulty ILRs, or already have a cardiac device were not included in the study. Unexplained syncope was defined as a transient loss of consciousness with full spontaneous recovery in which no definitive cause was identified after initial evaluation, including history, examination, 12-lead ECG, and, where appropriate, echocardiography, ambulatory monitoring, and tilt testing.

Patients were implanted with Medtronic Reveal LINQ ILRs (Dublin, Ireland). These provide continuous cardiac rhythm monitoring for about three years. They are equipped with wireless remote monitoring, allowing for automatic data uploads and alerts to physicians. Patients also have the ability to manually activate recording when they feel symptomatic.

Baseline demographics and clinical characteristics, including age, sex, cardiovascular risk factors, comorbidities, medication use, and results of prior investigations, were extracted from the hospital electronic records. The ILR findings were reviewed mostly from device interrogation reports on the electronic patient record. 

Device programming was standardised according to institutional protocols, with the automatic detection of bradyarrhythmia, tachyarrhythmia, and asystolic episodes. Stored rhythm strips triggered by patient activation or automatic detection were reviewed independently by a cardiac physiologist. Subsequently, all abnormal tracings were verified and adjudicated by a consultant cardiologist to determine their clinical significance. Indications for the insertion of cardiac implantable electronic devices (CIEDs) were based on the European Society of Cardiology (ESC) guidelines [5].

The primary outcome is the number of CIEDs implanted as a direct result of ILR findings. Secondary outcomes were as follows: (1) frequency of clinically significant events (CSEs), defined as symptomatic arrhythmia, asymptomatic arrhythmia judged relevant to the syncopal history, or the absence of arrhythmia during syncope; (2) comparison of baseline characteristics between patients with and without ILR-detected arrhythmias; and (3) complications related to ILR insertion.

Any complications that could arise due to ILRs, such as infection, device erosion, or the need for an early explant, were also noted.

Baseline characteristics were compared between patients with and without arrhythmia. For continuous variables, normality was assessed. Since the distribution of continuous variables did not meet assumptions of normality, comparisons were performed using the Mann-Whitney U test. For categorical variables, the chi-squared test was applied. A p-value of <0.05 was considered statistically significant. All analyses were conducted using IBM SPSS Statistics for Windows, V. 28.0 (IBM Corp., Armonk, NY, USA).

## Results

A total of 215 patients had an ILR implanted for the evaluation of syncope during the study period. Of these, 43 patients (20.5%) subsequently required the implantation of a CIED based on the ILR documented findings.

When baseline characteristics were compared between patients who had an arrhythmia detected and those without arrhythmia detection, age was the only statistically significant characteristic, with older patients being more likely to have clinically relevant arrhythmias necessitating CIED implantation (Table 1).

**Table 1:** Baseline characteristics of the patients with and without arrhythmia Continuous variables are presented as mean ± SD and compared with the independent t-test (t-statistic reported). Categorical variables are presented as n (%) and compared with the chi-squared test (χ² reported). A p-value of <0.05 was considered statistically significant. CKD: chronic kidney disease; COPD: chronic obstructive pulmonary disease; CVA: cardiovascular accident; MI: myocardial infarction

Variable	Arrhythmia detected (n = 92)	Arrhythmia not detected (n = 123)	Test statistic	P-value
Age (years)	66.4 ± 16.7	56.7 ± 17.6	t = 4.0	<0.01
Female gender (%)	38 (41.3%)	60 (49.2%)	χ² = 1.33	0.25
Diabetes (%)	13 (14.1%)	24 (19.7%)	χ² = 1.11	0.29
COPD (%)	11 (12%)	15 (12.3%)	χ² = 0.01	0.94
CKD (%)	3 (3.3%)	10 (8.2%)	χ² = 2.13	0.14
Previous MI (%)	17 (18.5%)	36 (29.5%)	χ² = 3.55	0.06
Hypertension (%)	35 (38%)	57 (46.7%)	χ² = 1.65	0.20
Hyperlipidaemia (%)	32 (34.8%)	53 (43.4%)	χ² = 1.67	0.20
Heart failure (%)	10 (10.9%)	15 (12.3%)	χ² = 0.10	0.75
CVA (%)	9 (9.8%)	16 (13.1%)	χ² = 0.56	0.45
Epilepsy (%)	5 (5.4%)	8 (6.6%)	χ² = 0.12	0.73

Across the entire cohort, 103 patients (47.9%) experienced at least one CSE during follow-up (Table 2). Among these, symptomatic arrhythmias represented the most common presentation (45.6% of CSEs). Within this category, the most frequent arrhythmia noted was a sinus pause of greater than six seconds (39.1%), with atrial fibrillation being the second most common arrhythmia (35.9%). Other arrhythmias, although occurring less frequently, included tachy-brady syndrome, supraventricular tachycardia, and high-level atrioventricular (AV) blocks (Table 3).

**Table 2:** CSEs CSEs: clinically significant events

CSEs	Number of patients (%)
Symptomatic arrhythmia	47 (34.8%)
Clinically significant asymptomatic arrhythmia	45 (7.9%)
No arrhythmia detected at the time of syncope	11 (5.1%)
Total no. of CSEs	103 (47.9%)

**Table 3:** Arrhythmias detected on ILRs No.: number; SVT: supraventricular tachycardia; ILRs: implantable loop recorders

Arrhythmia found	No. of patients (%)	Symptomatic (%)	Asymptomatic (%)
Sinus pause >6 seconds	36 (16.7%)	22 (61.1%)	14 (38.9%)
Atrial fibrillation	33 (15.3%)	14 (42.4%)	19 (57.6%)
Tachy-brady syndrome	8 (3.7%)	2 (25%)	6 (75%)
SVT	8 (3.7%)	4 (50%)	4 (50%)
Mobitz type 2	4 (1.8%)	2 (50%)	2 (50%)
Complete heart block	3 (1.4%)	3 (100%)	0 (0%)
Total no. of arrhythmias	92 (46%)	47 (51.1%)	45 (48.1%)

No major complications necessitating early explantation were observed. Minor issues such as local erythema or discomfort were conservatively managed. ILRs remained in situ in all patients until the completion of the intended monitoring period or until diagnostic yield was achieved. In our cohort, the time to arrhythmia diagnosis ranged from one day to 1078 days, with a mean of 200 days. For symptomatic arrhythmias, the corresponding range was one day to 1202 days, with a mean of 215 days.

## Discussion

Syncope is a common medical symptom, affecting between 15% and 39% of the general population, with an annual incidence of 18.1-39.7 episodes per 1,000 patients [6]. Reflex syncope is by far the most prevalent cause [7], but identifying the reason for recurrent, unexplained syncope remains a clinical challenge and an ongoing pursuit. ILRs are practical tools for diagnosing unexplained syncope, and their diagnostic yield may have been previously undermined.

This study confirms the clinical value of ILRs in patients with unexplained syncope. Nearly half of the patients experienced a clinically significant arrhythmic event, and one in five ultimately required device implantation. These findings are consistent with meta-analyses reporting diagnostic yields around 44% and extend real-world evidence to a UK tertiary centre population [8].

The 20% rate of CIED implantation based on ILR findings in our study is slightly higher than some previously reported real-world intervention rates, such as the Dutch registry, which noted 12% pacemaker implantation at one year and 14.6% at three years [9]. Differences from our figures may reflect centre-specific populations, monitoring duration, and thresholds for pacing. Our recorded CSEs at 48% align well with a large meta-analysis, which demonstrated an overall diagnostic yield of ~44%, including arrhythmias, with a median time to diagnosis of approximately 134 days. This similarity highlights the significant usefulness of ILRs in detecting arrhythmogenic correlates in syncopal patients, affirming our findings in wider practice.

Our finding that sinus pauses of greater than six seconds were the most prevalent arrhythmia mirrors other studies. In the older populations (>65 years), documented ILR usage yielded high arrhythmia detection rates, particularly AV blocks and sinus pauses of greater than three seconds being the most common [10]. These observations suggest that longer sinus pauses, particularly in older individuals, are clinically relevant markers warranting further therapeutic consideration. 

Loop recorders, when paired with remote monitoring, have been associated with faster diagnosis and higher detection rates versus in-clinic follow-up. In a multicentre propensity-matched study, remote monitoring yielded up to a 2.5-fold increase in diagnostic rate and ~46-day reduction in time to diagnosis, particularly benefiting asymptomatic episodes. Hence, integrating remote monitoring with ILRs may shorten the path from symptom capture to therapy [11].

Randomised data (FRESH study) demonstrates that early ILR implantation outperforms conventional pathways of investigating syncope, with higher yield and lower healthcare costs, supporting guideline recommendations of earlier ILR implantation in selected unexplained syncopal patients [12-14]. Our rates of CSE and device implantation are consistent with a clinically meaningful impact when ILR is used upfront.

This study adds to the current UK and international literature by providing one of the largest single-centre, retrospective cohorts of ILR implantation for unexplained syncope, highlighting both diagnostic yield and therapeutic consequences in real-world NHS practice [15]. The demonstration that one in five patients proceeded to device implantation, coupled with the identification of age as a predictor of diagnostic success, provides novel insights that may refine patient selection and inform healthcare resource allocation [16].

This study's limitations include its single-centre, retrospective design, which may limit generalisability. Unrecorded patient variables, such as pre-implantation evaluations, could affect the diagnostic yield. Future studies would potentially explore temporal trends and variation by symptoms such as pre-syncope and syncope and age-stratify the outcomes.

## Conclusions

In this single-centre, retrospective study, ILR implantation in patients with unexplained syncope demonstrated a high diagnostic yield, identifying clinically relevant arrhythmias in nearly half of the patients and leading to CIED implantation in one-fifth. The predominance of bradyarrhythmias, especially prolonged sinus pauses, underscores the value of ILRs in unmasking intermittent conduction disease.

ILR use was safe, with no major complications observed. These findings support guideline recommendations for early ILR consideration in patients with recurrent unexplained syncope, particularly in older individuals.

Future multicentre, prospective studies incorporating health-economic evaluations are warranted to refine ILR-based care pathways.
